# Incomplete T-cell tolerance and epitope-specific responses to the NR1 subunit in NMDA receptor antibody-associated encephalitis

**DOI:** 10.1093/braincomms/fcag298

**Published:** 2026-08-05

**Authors:** Rachel L Brown, Adriana Albuquerque, Sharyn Thomas, Angelika Holler, Heather Angus-Leppan, Alessandro Sette, Cecilia Lindestam Arlehamn, Melanie Hart, Michael S Zandi, Angela Vincent, Hans J Stauss, Michael P Lunn, Emma C Morris

**Affiliations:** UCL Institute of Immunity and Transplantation, University College London, London NW3 2PP, UK; Department of Neuromuscular Diseases, UCL Queen Square Institute of Neurology, University College London, London WC1N 3BG, UK; National Hospital for Neurology and Neurosurgery, University College London Hospitals Foundation NHS Trust, London WC1N 3BG, UK; National Institute for Health and Care Research (NIHR) University College London Hospitals (UCLH) Biomedical Research Centre, London NW1 2PG, UK; UCL Institute of Immunity and Transplantation, University College London, London NW3 2PP, UK; UCL Institute of Immunity and Transplantation, University College London, London NW3 2PP, UK; UCL Institute of Immunity and Transplantation, University College London, London NW3 2PP, UK; Department of Clinical Neurosciences, Royal Free Hospitals NHS Foundation Trust, London NW3 2QG, UK; Center for Infectious Disease, Division of Vaccine Discovery, La Jolla Institute for Immunology, San Diego, CA 92037, USA; Center for Infectious Disease, Division of Vaccine Discovery, La Jolla Institute for Immunology, San Diego, CA 92037, USA; Centre for Vaccine Research, Department of Infectious Disease Immunology, Statens Serum Institut, 5 Artillerivej, DK-2300 Copenhagen S, Denmark; Department of Neuroinflammation, UCL Queen Square Institute of Neurology, University College London, London WC1N 3BG, UK; National Hospital for Neurology and Neurosurgery, University College London Hospitals Foundation NHS Trust, London WC1N 3BG, UK; Department of Neuroinflammation, UCL Queen Square Institute of Neurology, University College London, London WC1N 3BG, UK; Nuffield Department of Clinical Neurosciences, John Radcliffe Hospital, University of Oxford, Oxford OX3 9DU, UK; UCL Institute of Immunity and Transplantation, University College London, London NW3 2PP, UK; Department of Neuromuscular Diseases, UCL Queen Square Institute of Neurology, University College London, London WC1N 3BG, UK; National Hospital for Neurology and Neurosurgery, University College London Hospitals Foundation NHS Trust, London WC1N 3BG, UK; UCL Institute of Immunity and Transplantation, University College London, London NW3 2PP, UK; National Institute for Health and Care Research (NIHR) University College London Hospitals (UCLH) Biomedical Research Centre, London NW1 2PG, UK; Department of Clinical Neurosciences, Royal Free Hospitals NHS Foundation Trust, London NW3 2QG, UK; Department of Clinical Haematology, University College London Hospitals Foundation NHS Trust, London NW1 2PG, UK

**Keywords:** NMDA receptor, T cells, immune tolerance

## Abstract

NMDA receptor antibody-associated (NMDAR-Ab) encephalitis primarily affects young women and children and often requires prolonged intensive care admission. Currently available first- and second-line therapies are effective, but patients can be refractory and residual deficits are not uncommon. Although B-cell lineages and specific NMDAR (NR1 subunit) antibodies have been examined in detail, the subtypes and roles of antigen-specific T cells in providing T-cell help to drive the IgG antibody response, cytotoxic effects or other roles have not been reported. Peripheral blood mononuclear cells from eight patients and eight healthy volunteers underwent three rounds of stimulation with pooled 15-mer peptides spanning the length of the NR1 sequence. At each round of stimulation, functional CD4+ and CD8+ T-cell responses to NR1-derived peptide pools were assessed using flow cytometry. CD4+ and CD8+ T responses to NR1-derived peptide pools were identified in both patients and healthy volunteers. Patient CD4+ T-cell responses were more frequent compared to healthy volunteers after the first round of stimulation, with fewer responses after the third round. Healthy volunteer CD4+ T cells showed the opposite trend (*P* = 0.004, *χ*² test for trend). Patient CD8+ T-cell responses were also stronger than healthy volunteers after just one round of stimulation, but not thereafter. Surprisingly, both patient and healthy volunteer CD8+ T cells responded more strongly to NR1-peptide pools than CD4+ T cells. Epitope mapping after round 3 identified two NR1 peptide epitopes as targets for CD4+ T cells and eight peptide epitopes as targets for CD8+ T cells. These epitopes were distributed throughout the NR1 sequence and were distant from the well-established antibody epitope. Although the kinetics of the responses to NR1 peptides in patients may be compatible with *in vivo* priming of autoreactive T cells or their reduced regulation, overall, the difference between patient and healthy volunteer responses was modest. Further investigation is needed to better characterize these responses and determine their implications for disease pathogenesis, prognosis and therapy. The existence of natural responses to NR1 as a potential autoantigen, particularly in the CD8+ T-cell lineage, should also be explored.

## Teaser

Brown *et al.* demonstrate the T-cell responses to NR1-derived peptides in patients with NMDA receptor antibody-associated encephalitis and healthy individuals. Surprisingly, CD8+ T-cell responses were more common than CD4+ responses. Immunogenic epitopes identified spanned the whole NR1 protein. These data join an emerging literature on T cells in autoimmune encephalitis and natural immunity to self-antigens.

## Graphical Abstract

**Graphical Abstract:**
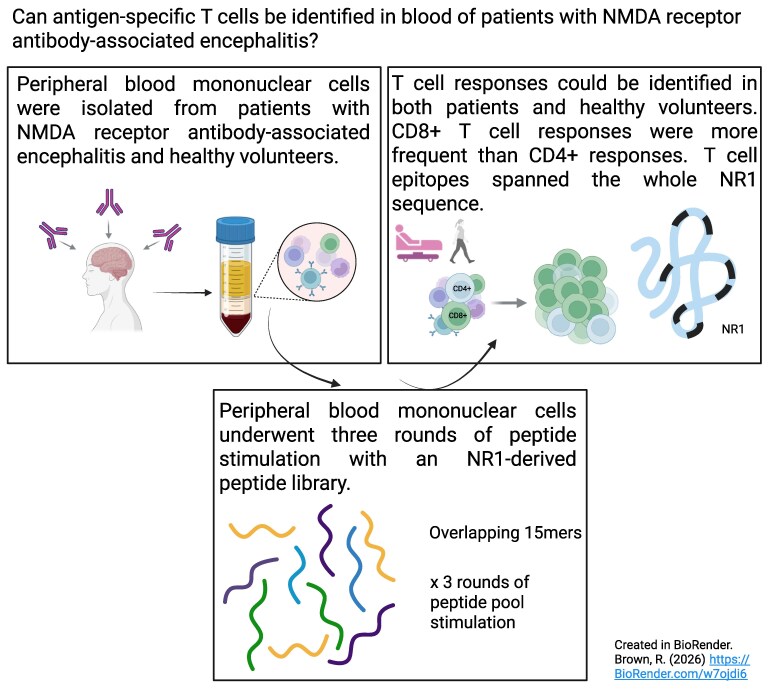

## Introduction

**Figure 1:**
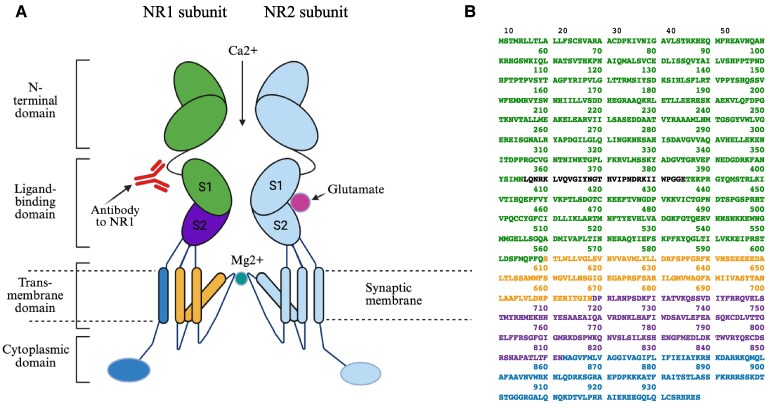
**Structure of the NMDA receptor.** (**A**) Schematic representation of the structure of the NMDA receptor (adapted from Gallo *et al.*^2^) and (**B**) NR1 sequence (sequence thought to contain the critical epitope for antibody binding and used in animal models of disease highlighted in black^3,4^) (https://www.uniprot.org/uniprotkb/Q05586/entry). Amino acid sequences in **B** correspond by colour to the structural subunits in **A**.

N-methyl-D-aspartate receptor (NMDAR) antibody encephalitis is predominantly a disease of young women and children and associated with antibodies to the NR1 subunit of the NMDAR (Fig. 1A).^1^ Classically, patients present with neuropsychiatric symptoms, seizures, complex movement disorders and autonomic dysfunction.^1^ There is often a need for prolonged intensive care admission and rehabilitation.^5,6^ Current first- and second-line therapies predominantly target the antibody and B-cell components of the disease, but many patients remain treatment refractory or experience long-term sequelae.^7,8^ There is a need to understand better the underlying immunopathology with the possibility of identifying new treatment targets or strategies.

Autoimmunity results from loss of B- and/or T-cell tolerance to self, leading to the production of autoantibodies or the presence of autoreactive T cells. The role of B-cell specificity and antibody effector mechanisms in NMDAR-Ab encephalitis is well established.^3,9,10^ By contrast, little is known about antigen-specific T-cell responses which may provide T-cell help or cytotoxic effector responses in this disease. Only one study focussing on T-cell specificity has been conducted in patients, using an antigen-rich T-cell enrichment assay, in which peripheral blood mononuclear cells (PBMCs) underwent a 7-h stimulation with either whole NR1 protein or NR1 protein fragments; this reported, surprisingly, reduced T-cell responsiveness to the NMDAR in patients compared with healthy volunteers.^11^ Recent animal models have shown the presence of NR1-specific T cells in spleen and meninges following immunization with short peptides spanning the B-cell epitope of the NR1 subunit.^4^ As T cells respond to peptide presented on major histocompatibility complex (MHC) molecules rather than conformational epitopes, and due to the principle of linked recognition, it is known that T-cell epitopes and B-cell epitopes do not need to overlap.^12,13^ Thus, there is a need for a more systematic investigation of antigen-specific T-cell responses in this disease.

We asked whether functional NR1-specific CD4+ and/or CD8+ T cells could be identified in PBMCs isolated from patients with NMDAR antibody encephalitis and whether these differed from healthy volunteers. We used an established method for expanding antigen-specific T cells, stimulating with pools of NR1-derived 15-mer overlapping peptides spanning the entire NR1 subunit. After the identification of T-cell responses to peptide pools, we used a matrix method to identify the T cell–recognized peptide epitopes. Patients and control PBMCs were studied in parallel. Human leukocyte antigen (HLA) prediction tools were used to identify potential HLA-peptide binding combinations and confirm the potential relevance of the experimental findings.

## Materials and methods

### Standard protocol approvals, registrations and patient consents

The study was approved by the South Central – Oxford C Ethics Committee (REC reference 17/SC/0494, IRAS 223833). Informed written consent or assent was provided by all participants or appropriate consultees in accordance with the study protocol. Participants were included if they had received a diagnosis of NMDAR antibody encephalitis at any time point or had been demonstrated to have NMDAR antibodies following herpes simplex virus (HSV) encephalitis. Previous immunotherapy was permitted, but patients receiving concurrent immunosuppressive medication were excluded.

### Peripheral blood mononuclear cell isolation and storage

PBMCs were isolated from EDTA blood samples by density gradient centrifugation and washed twice in phosphate-buffered saline (PBS). Isolated cells were cryopreserved in 90% foetal calf serum (FCS) and 10% dimethyl sulfoxide (DMSO) at −80°C prior to use, except in patient 8, where freshly isolated PBMCs were used in initial peptide stimulation and cryopreserved cells used as autologous feeders.

### Peptides

Overlapping 15-mer peptides were synthesized by Mimotopes Pty Ltd (Clayton, Victoria, Australia), spanning the extracellular (172 peptides) and intracellular (65 peptides) portions of the NR1 protein (Fig. 1B; Supplementary Fig. 1). Peptides were resuspended in DMSO at a stock concentration of 5 mM (extracellular) or 10 mM (intracellular) and stored at −20°C prior to use. Peptides were pooled into eight pools of 21 or 22 peptides (peptides spanning the extracellular domains of the NR1) and two pools of 32 or 33 peptides (peptides spanning the intracellular domains of the NR1) (Supplementary Fig. 1). Epstein–Barr virus (EBV) and cytomegalovirus (CMV) class I and II peptides gifted by the La Jolla Institute (A.S. and C.L.A.) were used as positive controls.^14^

### *In vitro* peptide pool stimulations

*In vitro* peptide stimulations were performed as per the protocol shown in Supplementary Fig. 2A. PBMCs were cultured in RPMI 1640 supplemented with 1% L-glutamine, 1% penicillin plus streptomycin and 10% heat inactivated FCS and cytokines IL2 (Roche) 10–20 U/mL, IL7 (R&D Systems) 5 ng/mL and IL15 (R&D Systems) 5 ng/mL. Cryopreserved PBMCs were thawed, washed twice and plated in 24 well plates, with 2 × 10^6^ cells/well in 2 mL culture medium. PBMCs were stimulated with peptide pools at a concentration of 0.5 µM per NR1 peptide, or 0.5 µg/mL for control CMV/EBV peptides. After 7 days, PBMCs were restimulated with the same peptide pools as per round 1, in the presence of irradiated (35 Gy) autologous feeders at a 1:1 ratio. In total, three 7-day rounds of peptide stimulation were completed per patient or healthy volunteer.

### Intracellular cytokine staining

At the end of each round of restimulation, an aliquot of cells was assessed for NR1 peptide-specific responses. Cells sampled at each stage were incubated with irradiated (35 Gy) autologous feeders at a 1:1 ratio for 18 h in a 96 well plate, with either peptide pools or DMSO only (negative ‘no peptide’ control) in the presence of Brefeldin A. T-cell responses to NR1-derived peptides were quantified by flow cytometry enumerating the percentage of gated cells producing IFNγ.

### Matrix approach for identifying immunogenic peptides

Matrices (method adapted from Betts *et al.*^15^) contained nine subpools for peptide pools 1–8 (22–23 peptides per pool) or 11 subpools for peptide pools 9 and 10 (32–33 peptides per pool). Subpools corresponded with peptides contained in either one row or column of the matrix grid (Supplementary Fig. 3). T cells remaining after the three rounds of NR1 peptide pool stimulation were stimulated with matrix subpools in the presence of autologous irradiated feeders. In addition, ‘no-peptide’ and ‘whole peptide pool’ stimulations were repeated to ensure the initial response could be replicated. The matrix grid was used to identify single or multiple immunogenic peptides based on the cross referencing of subpools eliciting T-cell responses. Where insufficient T cells remained to proceed with the whole matrix, a limited matrix was used, and T cells were tested with subpools corresponding to rows of the matrix only.

Following the identification of single immunogenic peptides, and where sample availability permitted, a further aliquot of PBMCs underwent three rounds of single-peptide stimulation using the identified immunogenic peptide. Culture conditions were as for the peptide pool stimulations. After three rounds of single-peptide stimulation, T cells were stimulated overnight with relevant or irrelevant peptides prior to intracellular cytokine staining.

### Flow cytometric analysis

T cells were stained with eBioscience Fixable Viability Dye eFluor 780 in PBS (dilution 1:1000) for 15 min at 4°C in the dark, then washed in PBS. Extracellular staining for T-cell surface antigens was performed using CD3-Pe-Cy7 (clone SK7, Biolegend), CD4-eFlour 450 (clone OKT-4, eBioscience) and CD8-FITC (clone G42-8, BD) at a dilution of 1:80 in PBS/1% FCS (FACS buffer), for 30 min at 4°C in the dark. Stained T cells were washed with FACS buffer then fixed for 20 min, washed in perm buffer, then permeabilized in perm buffer for 20 min at 4°C using BD Cytofix/Cytoperm Kit. Intracellular cytokine staining was performed using IFNγ-APC (clone B27, BD) at a dilution of 1:60 in perm buffer, for 40 min at 4°C in the dark. T cells were washed in perm buffer and resuspended in FACS buffer prior to analysis.

Flow cytometry was performed a BD LSRFortessa (5L SORP). Data analysis was performed using FlowJo v10.7.2 for Mac OS X. A standard gating strategy was used for all data analysis (as shown in Supplementary Fig. 2B).

### Human leukocyte antigen predictions

Peptide-MHC class I binding predictions were performed using NetMHCpan-4.1 (https://services.healthtech.dtu.dk/services/NetMHCpan-4.1/) and class II predictions using NetMHCIIpan-4.0 (https://services.healthtech.dtu.dk/services/NetMHCIIpan-4.0/).

Data inputs for searches were as follows:

1. Peptide sequences in FASTA format representing the amino acid sequence of immunogenic single or overlapping 15-mers identified using peptide matrices
2. The peptide length for use in predictions (9-mers for class I predictions and 15-mers for class II predictions)
3. HLA sequences for relevant patients or healthy volunteers for each peptide

Results displayed relate to predictions based on eluted ligand (EL) data. Criteria for strong binders (SB) and weak binders (WB) were based on % Rank EL (i.e. the percentile of the predicted binding affinity calculated by comparing the distribution of affinities calculated using a set of random natural peptides, and the standard setting for the prediction tool). For class I, the criteria were for SB < 0.5% and WB < 2% and for class II, % Rank SB < 1% and WB < 5%.

### Data analysis and presentation

Data analysis was completed in Microsoft Excel version 16.66.1 for MacOS. *χ*² test for trend was used to analyse compared frequency of T-cell responses over three rounds of peptide stimulation with NR1-derived peptide pools in patients and healthy volunteers. Analyses and graphical data were generated using GraphPad Prism, 10.2.0 for MacOS.

## Results

### Patients

PBMC from eight patients (eight female) and eight healthy volunteers (seven female, one male) were studied (Table 1). The median age of the patients and healthy volunteers was 36 years (range 17–50) and 33.5 years (range 23–43), respectively. Two patients had a teratoma and one had NMDAR antibodies following HSV encephalitis. Overall, the patient cohort was largely recovered, and median time from symptom onset to sample collection was 27 months (range 8–39). By the time of testing, three patients had become seronegative for NMDAR antibodies.

**Table 1:** Summary of demographics and clinical details for patients and healthy volunteers tested

Patient/healthy volunteer	P1	P2	P3	P4	P5	P6	P7	P8
Age at sampling (decade)	Fifth	Fourth	Third	Second	Fourth	Second	Fourth	Fifth
Clinical phenotype	Seizures, sleep disturbance, autonomic disturbance	Neuropsychiatric symptoms, seizures, movement disorder	Neuropsychiatric symptoms	Neuropsychiatric symptoms, seizures, dysautonomia	Neuropsychiatric symptoms, movement disorder, dysautonomia	Neuropsychiatric symptoms, seizures, movement disorder, dysautonomia	Neuropsychiatric symptoms, seizures, movement disorder	Herpes simplex virus encephalitis, seizures, confusion
Trigger		Teratoma			Teratoma			HSVE
CSF Ab at presentation	Positive	Positive	Positive	Positive	Positive	Positive	Positive	Unknown
Previous treatment given at point of sample collection	None	Steroids, PLEX, rituximab, cyclophosphamide, bortezomib, teratoma resection	Steroids	Steroids, IVIG	Steroids, PLEX, teratoma resection	Steroids, PLEX, rituximab	Steroids, PLEX	None
Months at sampling	11	33	8	24	30	15	39	36
Serum Ab at sample	Positive	Negative	Negative	Positive	Negative	Positive	Positive	Positive
Status at sampling	Symptoms ongoing	Recovered	Recovered	Recovered, new symptoms	Recovered	Improving	Recovered	Recovered

Ab, antibody; CSF, cerebrospinal fluid.

### NR1-specific autoreactive T-cell responses were identified in healthy volunteers and patients

After each 7-day round of *in vitro* stimulation, flow cytometry was used to identify CD4+ and CD8+ T-cell responses following overnight stimulation with either NR1 peptide pools in the presence of irradiated autologous feeders or irradiated autologous feeders only (‘no-peptide’ controls) (Supplementary Fig. 2A). A positive response to NR1-derived peptides was defined by meeting both of the following criteria:

1. Percentage of cells producing IFNγ in response to peptide were greater than the mean + 2 SD of all no-peptide control responses across all peptide pools tested for that individual
2. Percentage of cells producing IFNγ in response to peptide were >1.5× the % cells producing IFNγ in the corresponding no-peptide control response (i.e. a stimulation index > 1.5).

For healthy volunteer 2 in rounds 1 and 2 of stimulation, due to the absence of a ‘no peptide’ control, responses were compared to an unstimulated control (PBMCs which had not been stimulated with peptide at any stage).

**Figure 2:**
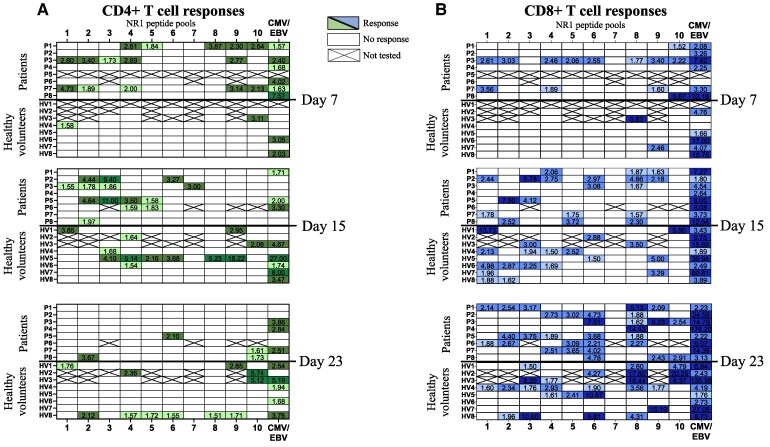
**Map of CD4+ T cell (A) and CD8+ T (B) responses to NR1-derived peptide pools, as measured by flow cytometry and following intracellular cytokine staining for IFNγ, over three rounds of peptide pool stimulation.** Green/blue coloured boxes indicate pools to which responses were identified. Numbers within coloured boxes indicate the stimulation index (SI) calculated by dividing the % cells producing IFNγ in response to peptide pools by the % producing IFNγ in the corresponding ‘no peptide’ control. Light green/blue boxes indicate SI 1.5–2, mid green/blue boxes indicate SI 2–5, and dark green/blue boxes indicate SI > 5. Blank boxes indicate no response identified (response criteria not met). Hatched box indicates sample not tested. P1–8, patients 1–8; HV1–8, healthy volunteers 1–8; CMV/EBV, cytomegalovirus/Epstein–Barr virus peptides.

Example responses of CD4 and CD8 T cells are shown in Supplementary Fig. 2B. NR1-specific autoreactive CD4+ T-cell IFNγ secretion was identified in both patients and healthy volunteers (Fig. 2A; Supplementary Table 1). At the earliest time-point, CD4+ T-cell responses were identified in 3/7 patients compared to 2/7 healthy volunteers (Fig. 2A). After two rounds of stimulation, NR1-specific CD4+ T cells were detectable in 5/8 patients and 6/8 healthy volunteers. Responses declined in patients after three rounds of peptide stimulation, while they were maintained in health volunteers. When analysed according to the number of different peptide pools recognized, there was a significant difference between the trend of responses in patients and healthy volunteers over the three rounds of stimulation (*P* = 0.004, *χ*² for trend).

CD8+ T-cell IFNγ secretion in response to NR1-derived peptide pools was also identified in both patients and healthy volunteers (Fig. 2B; Supplementary Table 1). Early CD8+ T-cell responses after one round of stimulation were seen in 4/7 patients and in 2/7 healthy individuals. Both patients and healthy volunteers mounted NR1-specific CD8+ T-cell responses after two rounds of peptide stimulation, and, unlike with CD4+ T-cell responses, CD8+ T-cell responses in both patients and healthy volunteers were frequently observed following the third round of peptide stimulation on day 23. These late CD8+ T-cell responses were most frequently seen in response to NR1-peptide pools 6 and 8 (Fig. 2B) in both patients and healthy volunteers, suggesting that these pools may contain immunodominant CD8+ T-cell epitopes.

### Peptide matrices identified specific immunodominant NR1 peptides

Where sufficient T cells remained after three rounds of peptide stimulation, and where a response to a peptide pool had been identified, a peptide matrix was used to identify individual immunogenic CD4+ and CD8+ peptide epitopes within the peptide pool. An example of a peptide matrix, corresponding to NR1 peptide pool 6, is shown in Supplementary Fig. 3. In this example, peptide-specific T-cell responses were identified using subpools N3 (column) and N8 (row), identifying peptide 121, amino acid sequence 499–513 of the NR1 protein (*NGMMGELLSGQADMI*), as the T cell–recognized peptide, designated as CD8+ epitope 4.

**Figure 3:**
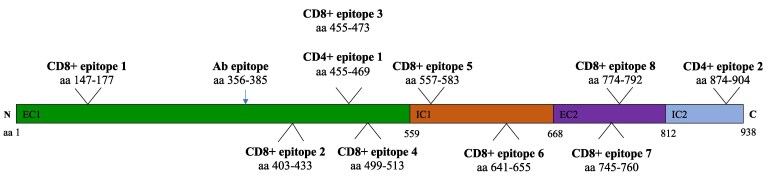
**Schematic representation of immunogenic peptide epitopes identified from matrix experiments performed after three rounds of NR1 peptide pool stimulation, mapped onto the structure of the NR1 protein.** aa, amino acid; EC1, first extracellular domain; IC1, transmembrane and first cytoplasmic domain; EC2, second extracellular domain; IC2, transmembrane and second cytoplasmic domain.

Overall, the T-cell epitopes identified spanned the breadth of the NR1 protein. In keeping with the greater number of CD8+ T-cell responses to peptide pools after three rounds of peptide pool stimulation compared to CD4+ T-cell responses, more CD8+ epitopes were identified using peptide matrices compared with CD4+ T-cell epitopes (Fig. 3; Supplementary Table 2). Within the CD4+ T-cell compartment, amino acids 874–904 of the NR1 protein contained CD4+ T-cell epitope 2, which was recognized by one patient and one healthy volunteer. Within the CD8+ T-cell compartment, eight immunogenic peptide epitopes were identified. NR1 amino acids 455–473 contained CD8+ T-cell epitope 3, which was recognized by two patients and one healthy volunteer. Similarly, amino acids 774–792 of the NR1 protein contained CD8+ T-cell epitope 8, which was recognized by one patient and one healthy volunteer. Responses to CD8+ T-cell epitopes 2, 5 and 6 were identified in one patient each, whereas epitope 4 generated a response in one healthy volunteer.

A more limited matrix (using smaller peptide pools corresponding to matrix rows) identified CD8+ T-cell responses to sequences contained within regions spanning amino acids 147–177 (CD8+ T-cell epitope 1) in three different patients and amino acids 403–443 (CD8+ T-cell epitope 2) in one patient.

### Single-peptide expansions confirmed predictions from matrix experiments

To determine whether the matrix method had correctly identified immunogenic peptides, we performed *in vitro* stimulations using single peptides in a patient and healthy volunteer that had sufficient cells remaining after the initial screening protocol. Matrix experiments identified responses to CD8+ T-cell epitope 3 (*CYGFCIDLLIKLARTMNFT*) in one patient and one healthy volunteer. After three rounds of stimulation with a single 15-mer peptide from within this epitope (*CYGFCIDLLIKLART*), flow cytometric analysis identified CD8+ T cells which secreted IFNγ in response to relevant but not irrelevant peptide (Supplementary Fig. 4A and C). Additionally, a population of IFNγ secreting CD4+ T cells in response to *CYGFCIDLLIKLART* peptide stimulation was identified in the same patient and healthy volunteer (Supplementary Fig. 4B and D).

### Human leukocyte antigen binding predictions

We undertook a bioinformatics approach to predict which patient or healthy volunteer HLA molecules may have presented the identified immunogenic peptides. HLA binding predictions were made for the identified immunogenic epitopes using HLA haplotypes of the patients or healthy volunteers in whom responses to those epitopes were identified. For all identified epitopes, with the exception of epitope 4 which was recognized by healthy volunteer 5, at least one relevant peptide-HLA binding prediction met strong or weak binding criteria.

**Figure 4:**
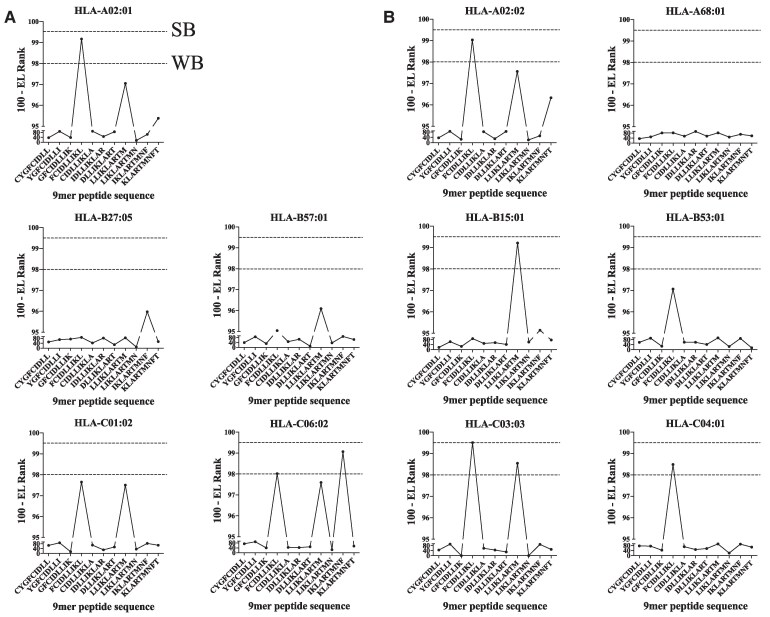
**HLA binding predictions for identified immunogenic epitopes.** Class I binding predictions for CD8+ T-cell epitope 3 in patient 8 (**A**) and healthy volunteer 8 (**B**). Binding predictions were made using NetMHCpan-4.1 for class I using the identified epitopes and the known HLA molecules of the responding patients and healthy volunteers. Top dashed line represents cut-off for strong binding (SB) criteria; bottom dashed line represents cut-off for weak binding (WB) criteria. For visual presentation, peptide-MHC binding predictions for each peptide are presented using the bioinformatically calculated prediction score (% Rank EL) subtracted from 100% (used as the optimal score), i.e. 100 − % Rank_EL. EL, eluted ligand.

Examples of class I HLA binding predictions are given in Fig. 4A and B. Class I predictions were generated for the patient and healthy volunteer who demonstrated responses to CD8+ T-cell epitope 3 (*CYGFCIDLLIKLARTMNFT*). In the patient, it was predicted that 9-mer sequences contained within epitope 3 could be presented by HLA-A02:01 or HLA-C06:02. In the healthy volunteer, it was predicted that one 9-mer sequence (*FCIDLLIKL*) could be presented by HLA-A02:02, HLA-C03:03 or HLA-C04:01, and a second 9-mer sequence (*LLIKLARTM*) could be presented by HLA-B15:01 or HLA-C03:03.

Class II HLA binding predictions were generated for the healthy volunteer who demonstrated responses to CD4+ T-cell epitope 2 (Supplementary Fig. 5). Seven out of nine relevant HLA molecules (DRB1_0901, DRB5_0101, DQA10102-DQB10303, DQA10302-DQB10303, DRB1_1501, DQA10302-DQB10601 and DPA10202-DPB10501) were predicted to have sufficient binding strength to present 15-mer sequences from within this epitope, four meeting strong binding and three weak binding criteria.

## Discussion

NMDAR antibody-associated encephalitis has become the best-known antibody-mediated disorder of the CNS, but little is known about the underlying T-cell immunity, which is likely to exist to at least provide T-cell help to drive humoral responses. Using pools of overlapping NR1-derived peptides, we demonstrated that immunological tolerance to the NMDAR is incomplete in both patients and healthy volunteers. In the patients, who were mostly recovered or recovering from NMDAR encephalitis, CD4+ T-cell responses occurred more frequently after the first and second rounds of stimulation compared to after the third round, whereas the responses in the volunteers showed the opposite trend. CD8+ T cells, by contrast, were more common in patients after one round of peptide stimulation, but similar in both patients and volunteers after the second and third round of stimulation. Overall, the results indicate autoreactive potential in the peripheral CD4+ and CD8+ T-cell repertoire of both patients and healthy individuals in whom NR1-specific T-cell clones could be primed and expanded with repeated peptide stimulations and immunogenic epitopes mapped.

The early responses among the CD4+ and CD8+ T cells in patients may suggest either the presence of primed autoreactive NR1-specific T-cell clones in patients consistent with memory from previous exposure, or reduced regulation of T cells capable of recognizing NR1-derived peptides. However, conversely, as most patients in our study were recovered, it is also possible that the responses were influenced by survivorship bias. T-cell clones relevant to disease pathogenesis could have been depleted by immunotherapy and no longer present in the patients sampled, consistent with the absence of clinical symptoms even though some patients remained seropositive. Further work to compare responses at onset of disease and prior to immunotherapy is needed to try to differentiate these possible interpretations.

In patients and healthy volunteers, CD4+ and CD8+ T-cell responses were found in response to different peptide pools spanning all regions of the NR1 protein. Patients or healthy volunteers frequently demonstrated CD4+ and CD8+ T-cell responses to more than one region of the NR1 protein. This is in keeping with previous studies of T-cell responses to neuronal or glial proteins in other neurological autoimmune disorders, including narcolepsy and Guillain–Barré syndrome, demonstrating the importance of screening the whole protein sequence.^16,17^ The differences between mechanisms of antigen recognition by B cells and T cells, and the principle of linked recognition, mean that B cells can receive T-cell help from T cells recognizing different epitopes to those of the B cells, providing the T-cell epitope is from the same protein containing the B-cell epitope or a protein closely linked.^12,13^ A recent animal model of NMDAR encephalitis identified the presence of NR1-specific CD4+ and CD8+ T cells in spleen and meninges following immunization with the commonly accepted NR1 B-cell binding epitope contained within NR1 amino acids 358–380 (*RKLVQVGIYNGTHVIPNDRK*).^4^ The multiple epitopes identified in our study, which were all remote from this B-cell epitope, suggest that immunization models should be induced by the whole NR1 subunit rather than a single peptide.

Matrix experiments identified CD4+ and CD8+ T-cell epitopes that were able to elicit responses in more than one patient and/or healthy volunteer, suggesting that there may be specific regions of the NR1 protein that are particularly immunogenic. As matrix experiments were performed after three rounds of peptide stimulation and dependent upon sufficient cells remaining, it may be that there were additional epitopes present, in patients/pools where there were either insufficient cells remaining or responses at earlier rounds of stimulation. This may have included epitopes within or near the B-cell binding epitope. HLA binding predictions identified both HLA class I and class II alleles that are predicted to have sufficient binding strength to present peptides within the identified epitopes for recognition by the TCRs of CD8+ and CD4+ T cells, respectively. Further experimental validation will be necessary to determine whether these predictions are correct and if these peptides have a role in disease pathogenesis.

It was somewhat surprising that *in vitro* autoreactivity was greatest in the CD8+ T-cell compartment in both patients and healthy volunteers. More CD8+ T-cell responses were identified, and unlike in the CD4+ T-cell compartment, in both patients and healthy volunteers, responses persisted after three rounds of peptide stimulation. While CNS antigen-specific CD8+ T cells are presumed to have a pathogenic role in paraneoplastic neurological syndromes in which antibodies target intracellular antigens, CD8+ T cells have been little characterized in encephalitis syndromes in which antibodies targeting cell surface antigens are most clearly defined as pathogenic effectors. This fact by no means excludes a role for T cells. A recent clinical study examining T-cell subsets in CSF and blood suggested a correlation between a higher proportion of CD8+ T cells and poorer clinical outcomes, though the specificity of these cells was not determined.^18^ A further study using single-cell multi-omics showed an expansion of clonal CD8+ T cells in the blood and CSF of patients and suggested that activated CD8+ T cells may have a role in modulating B-cell function in this syndrome.^19^ Further work is needed to better understand whether NR1-specific autoreactive CD8+ T cells contribute to disease pathogenesis in humans and how they may influence clinical outcomes including duration of illness, presence of brain injury or atrophy or post-encephalitis epilepsy.

Our assays used repeated rounds of peptide stimulation. Similar expansion has been shown to be required in previous studies of neurological autoimmunity.^16^ This method has the advantage of being able to expand low-frequency autoreactive T-cell clones which may be relevant to the disease. However, prolonged culture limits interpretation of effector phenotype compared to immediate *ex vivo* analysis, as has been used in more recent studies.^20^ Further work to analyse responses in patients in the acute and recovery phase will help address these outstanding questions further.

The ability to expand NR1-specific T cells in healthy volunteers is of interest. Other studies using expanded T-cell libraries in central or peripheral nervous system autoimmune disease have often shown a lesser response to neuronal autoantigens in healthy volunteers compared to those in patients.^16^ Whether this difference is methodological, reflecting the use of repeated peptide stimulations to break tolerance (compared to, for example, non-antigen driven polyclonal expansion prior to peptide stimulation), or whether the NR1 protein is intrinsically immunogenic is unclear. Whether responses are present in healthy volunteers or not, the presence of autoreactive T cells alone is not sufficient to confirm pathogenicity. Further work must explore functional differences between autoreactive T cells and their regulation in patient and healthy volunteers to determine whether they may have a potential role in disease pathogenesis, or be important in treatment, outcomes and prognosis.

We recognize several important limitations of this study including small patient numbers and the duration between disease onset and sample collection in patients studied reflecting the rarity of suitable patients, the number of PBMCs required to complete experiments and the challenging experimental protocols. The patients studied were predominantly recovered, thus the presence and role of autoreactive T cells in the induction phase remains unknown. Patients also had different triggers for encephalitis, including ovarian teratoma, post-HSVE or unknown and the immune mechanisms involved in breaking tolerance in each case may have varied. Nonetheless, these patients collectively displayed a breakdown of tolerance to the NMDA receptor, and these late and natural responses may help us understand the factors involved in the initial breakdown of immune tolerance and the kinetics of immune memory following CNS autoimmune syndromes. We concentrated on IFNγ responses, which were a reliable discriminator between positive and negative responses in our cultures. However, this is just one marker of the T-cell response to peptide-specific stimulation and may be selective for specific T-cell subsets. Responses that may have been identified by measuring surface activation markers, cell division or production of other cytokines may have been missed. HLA binding predictions were not confirmed experimentally, and this is the subject of future study. This study focussed on T-cell responses in the blood and understanding the specificity of T cells in different compartments including CSF would aid understanding of the potential role of autoreactive T cells in both health and disease. This should also be a subject of future study.

However, these results provide important early insights into the breakdown of T-cell tolerance to the NR1 protein, a comparison of response profiles between patients and healthy individuals and the potential for relatively limited peptide epitopes (which are not restricted to sequences found within the B-cell epitope) in helping drive T-cell responses in NMDAR encephalitis.

## Supplementary Material

fcag298_Supplementary_Data

## Data Availability

Data are available on reasonable request to the corresponding author.
