# Supplementary material for: Incomplete T-cell tolerance and epitope-specific responses to the NR1 subunit in NMDA receptor antibody-associated encephalitis
Source: Brain Commun. 2026 Aug 5;8(4):fcag298. doi: 10.1093/braincomms/fcag298 (PMC13479385; doi:10.1093/braincomms/fcag298)
Supplement: Supplementary file 1 [file fcag298_supplementary_data.zip › Supplementary_Figures.pdf]

Incomplete T cell tolerance and epitope-specific responses to the NR1 subunit in NMDA receptor antibody-associated encephalitis

Supplementary material

EC1	EC2	IC1	IC2

Pool 1<br>a.a. 18-117	Pool 2<br>a.a.107-201	Pool 3<br>a.a. 191-289	Pool 4<br>a.a.278-373	Pool 5<br>a.a. 363-461	Pool 6<br>a.a. 450-545	Pool 7. a.a. 535-559,<br>+ SA, 668-728	Pool 8<br>a.a. 717-812	Pool 9<br>a.a. 1-31, 548-671	Pool 10<br>a.a. 802-938
RAACDPKIVNIGAVL	VSYTAGFYRIPVLGL	AEKVLQFDPGTKNVT	AHISDAVGVVAQAVH	QVGIYNGTHVIPNDR	VPQCCYGFCIDLLIK	YQGLTILVKKEIPRS	IQAVRDNKLHAFIWD	MSTMRLLTLALLFSC	SNAPATLTFENMAGV
DPKIVNIGAVLSTRK	AGFYRIPVLGLTTRM	LQFDPGTKNVTALLM	DAVGVVAQAVHELLE	YNGTHVIPNDRKIIW	CYGFCIDLLIKLART	TILVKKEIPRSTLDS	RDNKLHAFIWDSAVL	RLLTLALLFSCSVAR	ATLTFENMAGVFMLV
VNIGAVLSTRKHEQM	RIPVLGLTTRMSIYS	PGTKNVTALLMEAKE	VVAQAVHELLEKENI	HVIPNDRKIIWPGGE	CIDLLIKLARTMNFT	KKEIPRSTLDSFMQP	LHAFIWDSAVLEFEA	LALLFSCSVARAACD	FENMAGVFMLVAGGI
AVLSTRKHEQMFREA	LGLTTRMSIYSDKSI	NVTALLMEAKELEAR	AVHELLEKENITDPP	NDRKIIWPGGETEKP	LIKLARTMNFTYEVH	EIPRSTLDSFMQPFQ	IWDSAVLEFEASQKC	FSCSVARAACDPKIV	AGVFMLVAGGIVAGI
TRKHEQMFREAVNQA	TRMSIYSDKSIHLSF	LLMEAKELEARVIIL	LLEKENITDPPRGCV	IIWPGGETEKPRGYQ	ARTMNFTYEVHLVAD	KSKKRNYENLDQLSY	AVLEFEASQKCDLVT	VARAACDPKIVNIGA	MLVAGGIVAGIFLIF
EQMFREAVNQANKRH	IYSDKSIHLSFLRTV	AKELEARVIILSASE	ENITDPPRGCVGNTN	GGETEKPRGYQMSTR	NFTYEVHLVADGKFG	RNYENLDQLSYDNKR	FEASQKCDLVTTGEL	STLDSFMQPFQSTLW	GGIVAGIFLIFIEIA
REAVNQANKRHGSWK	KSIHLSFLRTVPPYS	EARVIILSASEDDAA	DPPRGCVGNTNIWKT	EKPRGYQMSTRLKIV	EVHLVADGKFGTQER	NLDQLSYDNKRGPKA	QKCDLVTTGELFFRS	SFMQPFQSTLWLLVG	AGIFLIFIEIAYKRH
NQANKRHGSWKIQLN	LSFLRTVPPYSHQSS	IILSASEDDAATVYR	GCVGNTNIWKTGPLF	GYQMSTRLKIVTIHQ	VADGKFGTQERVNNS	DRPEERITGINDPRL	LVTTGELFFRSGFGI	PFQSTLWLLVGLSVH	LIFIEIAYKRHKDAR
KRHGSWKIQLNATSV	RTVPPYSHQSSVWFE	ASEDDAATVYRAAAM	NTNIWKTGPLFKRVL	STRLKIVTIHQEPFV	KFGTQERVNNSNKKE	ERITGINDPRLRNPS	GELFFRSGFGIGMRK	TLWLLVGLSVHVVAV	EIAYKRHKDARRKQM
SWKIQLNATSVTHKP	PYSHQSSVWFEMMRV	DAATVYRAAAMLNMT	WKTGPLFKRVLMSSK	KIVTIHQEPFVYVKP	QERVNNSNKKEWNGM	GINDPRLRNPSDKFI	FRSGFGIGMRKDSPW	LVGLSVHVVAVMLYL	KRHKDARRKQMQLAF
QLNATSVTHKPNAIQ	QSSVWFEMMRVYSWN	VYRAAAMLNMTGSGY	PLFKRVLMSSKYADG	IHQEPFVYVKPTLSD	NNSNKKEWNGMMGEL	PRLRNPSDKFIYATV	FGIGMRKDSPWKQNV	SVHVVAVMLYLLDRF	DARRKQMQLAFAAVN
TSVTHKPNAIQMALS	WFEMMRVYSWNHIIL	AAMLNMTGSGYVWLV	RVLMSSKYADGVTGR	PFVYVKPTLSDGTCK	KKEWNGMMGELLSGQ	NPSDKFIYATVKQSS	MRKDSPWKQNVSLSI	VAVMLYLLDRFSPFG	KQMQLAFAAVNVWRK
HKPNAIQMALSVCED	MRVYSWNHIILLVSD	NMTGSGYVWLVGERE	SSKYADGVTGRVEFN	VKPTLSDGTCKEEFT	NGMMGELLSGQADMI	KFIYATVKQSSVDIY	SPWKQNVSLSILKSH	LYLLDRFSPFGRFKV	LAFAAVNVWRKNLQD
AIQMALSVCEDLISS	SWNHIILLVSDDHEG	SGYVWLVGEREISGN	ADGVTGRVEFNEDGD	LSDGTCKEEFTVNGD	GELLSGQADMIVAPL	ATVKQSSVDIYFRRQ	QNVSLSILKSHENGF	DRFSPFGRFKVNSEE	AVNVWRKNLQDRKSG
ALSVCEDLISSQVYA	IILLVSDDHEGRAAQ	WLVGEREISGNALRY	TGRVEFNEDGDRKFA	TCKEEFTVNGDPVKK	SGQADMIVAPLTINN	QSSVDIYFRRQVELS	LSILKSHENGFMEDL	PFGRFKVNSEEEEED	WRKNLQDRKSGRAEP
CEDLISSQVYAILVS	VSDDHEGRAAQKRLE	EREISGNALRYAPDG	EFNEDGDRKFANYSI	EFTVNGDPVKKVICT	DMIVAPLTINNERAQ	DIYFRRQVELSTMYR	KSHENGFMEDLDKTW	FKVNSEEEEEDALTL	LQDRKSGRAEPDPKK
ISSQVYAILVSHPPT	HEGRAAQKRLETLLE	SGNALRYAPDGILGL	DGDRKFANYSIMNLQ	NGDPVKKVICTGPND	APLTINNERAQYIEF	RRQVELSTMYRHMEK	NGFMEDLDKTWVRYQ	SEEEEEDALTLSSAM	KSGRAEPDPKKKATF
VYAILVSHPPTPNDH	AAQKRLETLLEERES	LRYAPDGILGLQLIN	KFANYSIMNLQNRKL	VKKVICTGPNDTSPG	INNERAQYIEFSKPF	ELSTMYRHMEKHNYE	EDLDKTWVRYQECDS	EEDALTLSSAMWFSW	AEPDPKKKATFRAIT
LVSHPPTPNDHFTPT	RLETLLEERESKAEK	PDGILGLQLINGKNE	YSIMNLQNRKLVQVG	ICTGPNDTSPGSPRH	RAQYIEFSKPFKYQG	MYRHMEKHNYESAAE	KTWVRYQECDSRSNA	LTLSSAMWFSWGVLL	PKKKATFRAITSTLA
PPTPNDHFTPTPVSY	LLEERESKAEKVLQF	LGLQLINGKNESAHI	NLQNRKLVQVGIYNG	PNDTSPGSPRHTVPQ	IEFSKPFKYQGLTIL	MEKHNYESAAEAIQA	RYQECDSRSNAPATL	SAMWFSWGVLLNSGI	ATFRAITSTLASSFK
NDHFTPTPVSYTAGF	RESKAEKVLQFDPGT	LINGKNESAHISDAV	RKLVQVGIYNGTHVI	SPGSPRHTVPQCCYG	KPFKYQGLTILVKKE	NYESAAEAIQAVRDN	CDSRSNAPATLTFEN	FSWGVLLNSGIGEGA	AITSTLASSFKRRRS
TPTPVSYTAGFYRIP		KNESAHISDAVGVVA		PRHTVPQCCYGFCID		AAEAIQAVRDNKLHA		VLLNSGIGEGAPRSF	TLASSFKRRRSSKDT
								SGIGEGAPRSFSARI	SFKRRRSSKDTSTGG
								EGAPRSFSARILGMV	RRSSKDTSTGGGRGA
								RSFSARILGMVWAGF	KDTSTGGGRGALQNQ
								ARILGMVWAGFAMII	TGGGRGALQNQKDTV
								GMVWAGFAMIIVASY	RGALQNQKDTVLPRR
								AGFAMIIVASYTANL	QNQKDTVLPRRAIER
								MIIVASYTANLAAFL	DTVLPRRAIEREEGQ
								ASYTANLAAFLVLDR	PRRAIEREEGQLQLC
								ANLAAFLVLDRPEER	IEREEGQLQLCSRHR
								AFLVLDRPEERITGI	REEGQLQLCSRHRES
								LDRPEERITGINDPR	

Supplementary figure 1: Amino acid sequences of NR1 peptide pools

aa = amino acid; EC1 = first extracellular domain; IC1 = transmembrane and first cytoplasmic domain, EC2 = second extracellular domain, IC2 = transmembrane and second cytoplasmic domain.

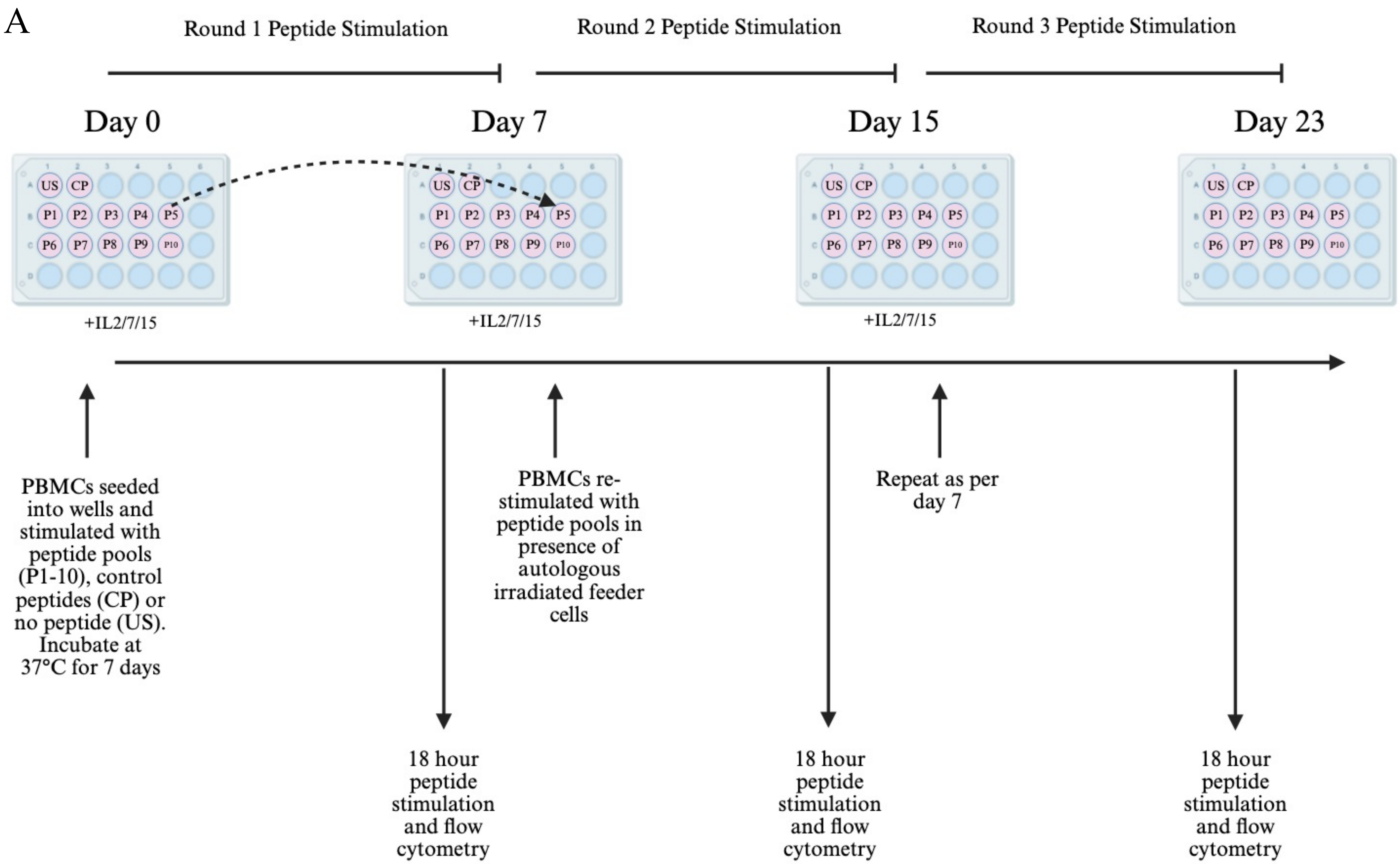

Supplementary Fig. 2A

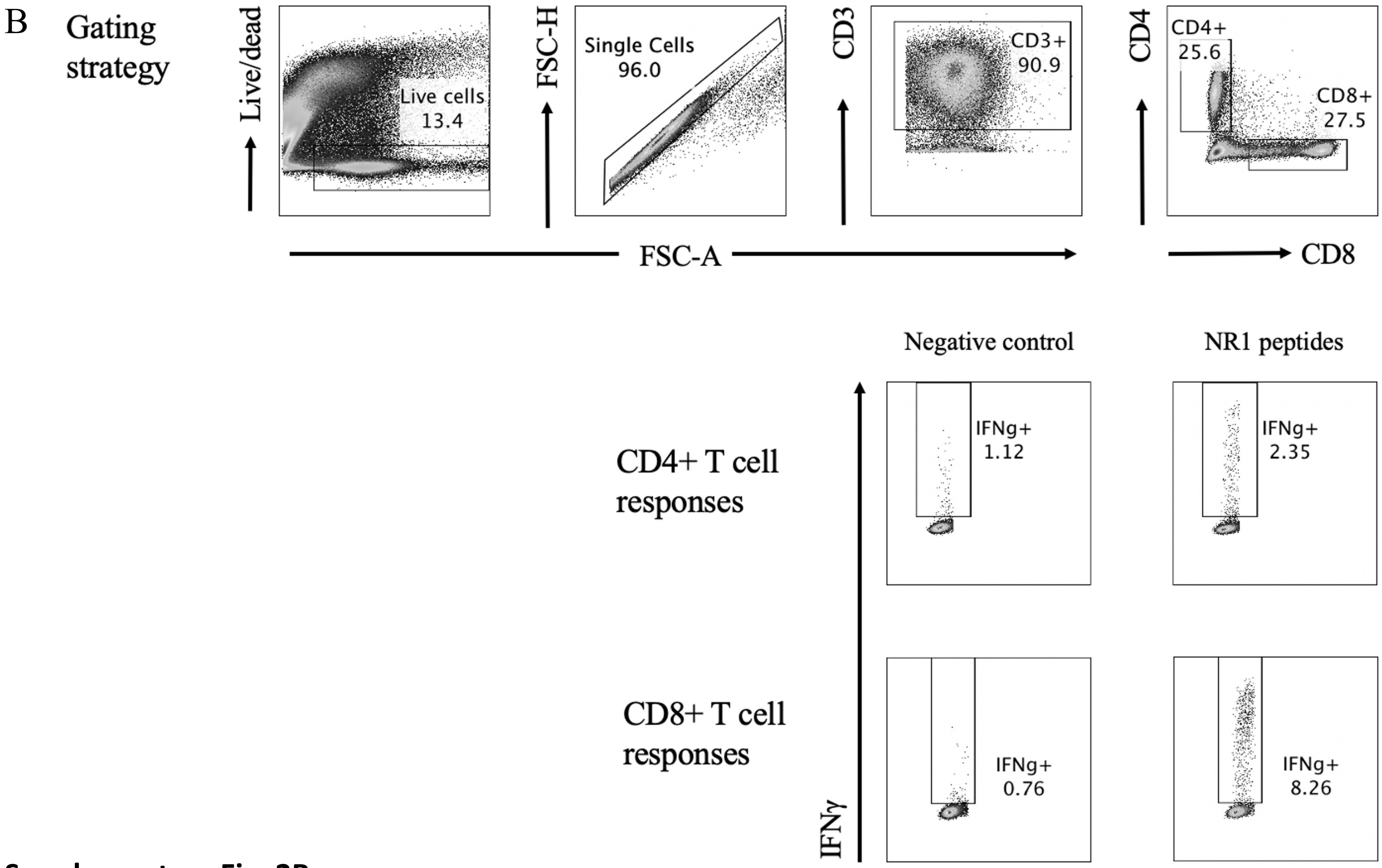

Supplementary Fig. 2B

Supplementary figure 2:

2a: Method for in vitro peptide pool stimulations. PBMCs were stimulated with peptide pools on day 0, and again at days 7 and 15 in the presence of autologous irradiated feeder cells. At the end of each round of stimulation, cells were sampled for overnight stimulation with peptide or no-peptide and analysed for responses by flow cytometry. Created in BioRender. Brown, R. (2026) https://BioRender.com/bt3rn33. CP = control (cytomegalovirus/Epstein Barr virus) peptide pool; PBMCs = peripheral blood mononuclear cells; P1-10 = peptide pools 1-10; US = unstimulated.

2b: Gating strategy used throughout analysis for determining % T cells producing IFNγ in response to peptide stimulation. Cells are gated on Live cells → Single cells → CD3+ T cells → CD4+ or CD8+ T cells. Representative examples of IFNγ responses – top row shows an example of a CD4+ response, comparing the negative 'no peptide' control (NPC, left hand plot) with response to peptide pool (right hand plot), bottom row shows an example of a CD8+ T cell response. In each panel, number indicates % of total cells falling within the designated gate. Both x and y axis are logarithmic scales. This gating strategy was used for all patients and healthy volunteers (total n = 16). FSC-A = forward scatter area; FSC-H = forward scatter height; IFNg = interferon gamma.

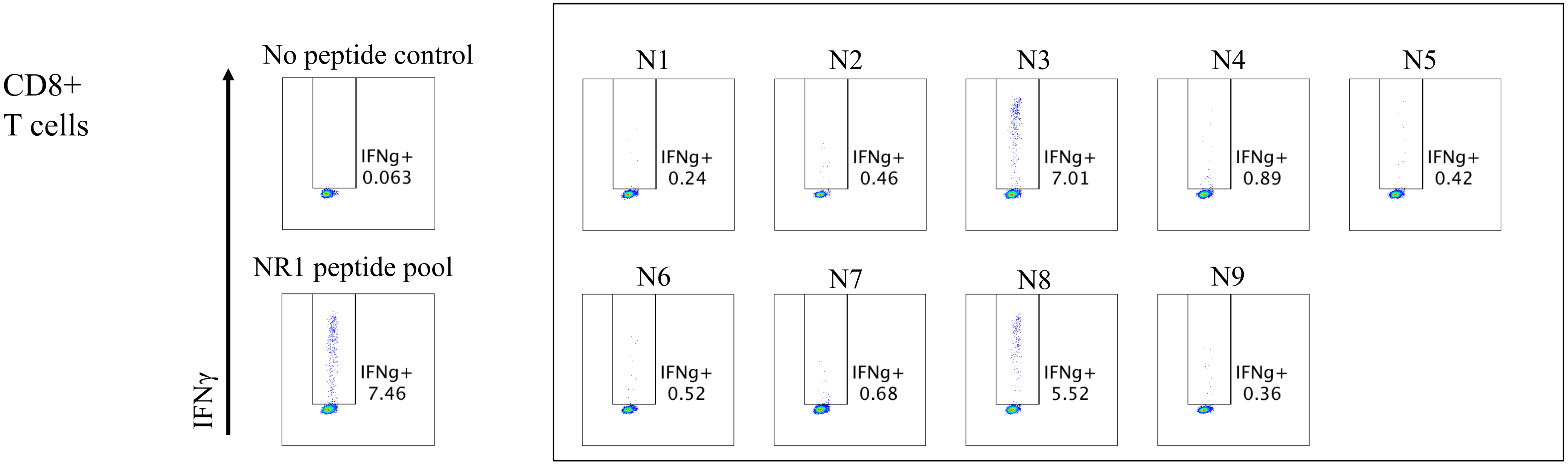

Epitope pool

Epitope pool	N1	N2	N3	N4	N5	% IFNγ+
N6	**109**	**110**	**111**	**112**	**113**	**0.52**
N7	**114**	**115**	**116**	**117**	**118**	**0.68**
N8	**119**	**120**	**121**	**122**	**123**	**5.52**
N9	**124**	**125**	**126**	**127**	**128**	**0.36**
	**129**	**129**				
% IFNγ+	**0.24**	**0.46**	**7.01**	**0.89**	**0.42**	

% IFNγ+

	NPC	Peptides
Pool 6	0.063	7.46

Immunogenic peptide = p121

Supplementary Fig. 3

Supplementary figure 3: Example of peptide matrix. Flow cytometry plots show % cells producing IFNγ in response to either no peptide ('no peptide control') or whole peptide pool (plots on left of box) or matrix subpools corresponding with peptides contained in columns or rows of the matrix grid (plots in box). Each number within the matrix grid represents a peptide from within the peptide pool. Adjacent peptides are overlapping by 11 amino acids. Due to the arrangement of the grid, each peptide is uniquely represented within one column/row pair. In this example, there is a response to subpools N3 (column 3) and N8 (row 3), thus peptide 121 is identified as the immunogenic peptide. The shown data are one example of n = 19 subpool analyses used to identify immunogenic peptide epitopes. IFNg = interferon gamma; NPC = no peptide control.

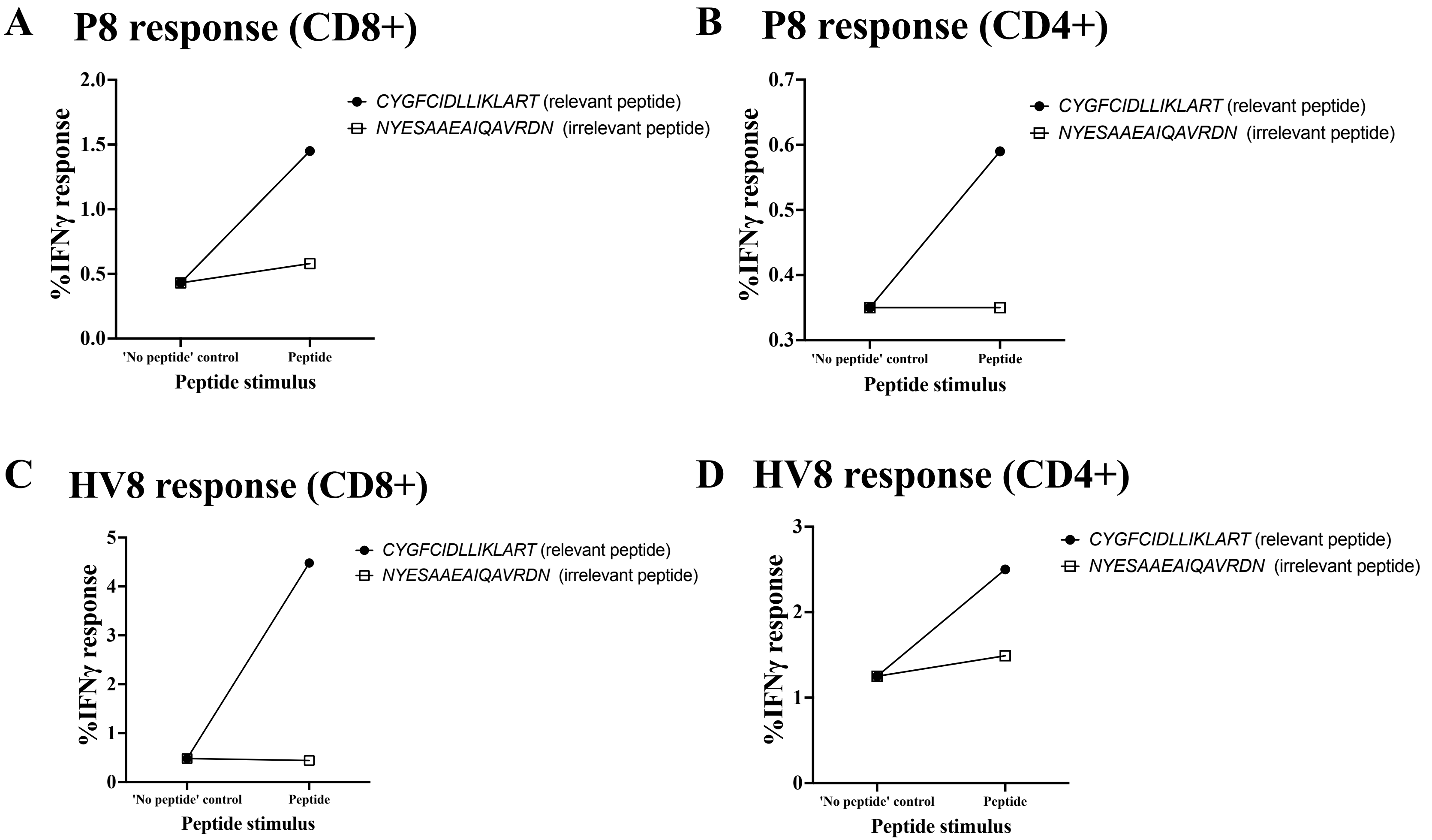

Supplementary figure 4A-D: Percentage of CD8+ or CD4+ T cells producing IFNγ as measured by flow cytometry in response to stimulation with single peptides. In A-D PBMCS from patient 8 (A and B) and healthy volunteer 8 (C and D) which have undergone three rounds of stimulation with single peptide amino acids 459-473 of the NR1 protein (*CYGFCIDLLIKLART*) underwent a final stimulation with either *CYGFCIDLLIKLART*, an irrelevant peptide, or no peptide (irradiated feeders only). Datapoints show % CD8+ (A and C) and CD4+ (B and D) T cells producing IFNγ following final stimulation with either *CYGFCIDLLIKLART*, irrelevant peptide or no peptide. HV8 = healthy volunteer 8; P8 = patient 8.

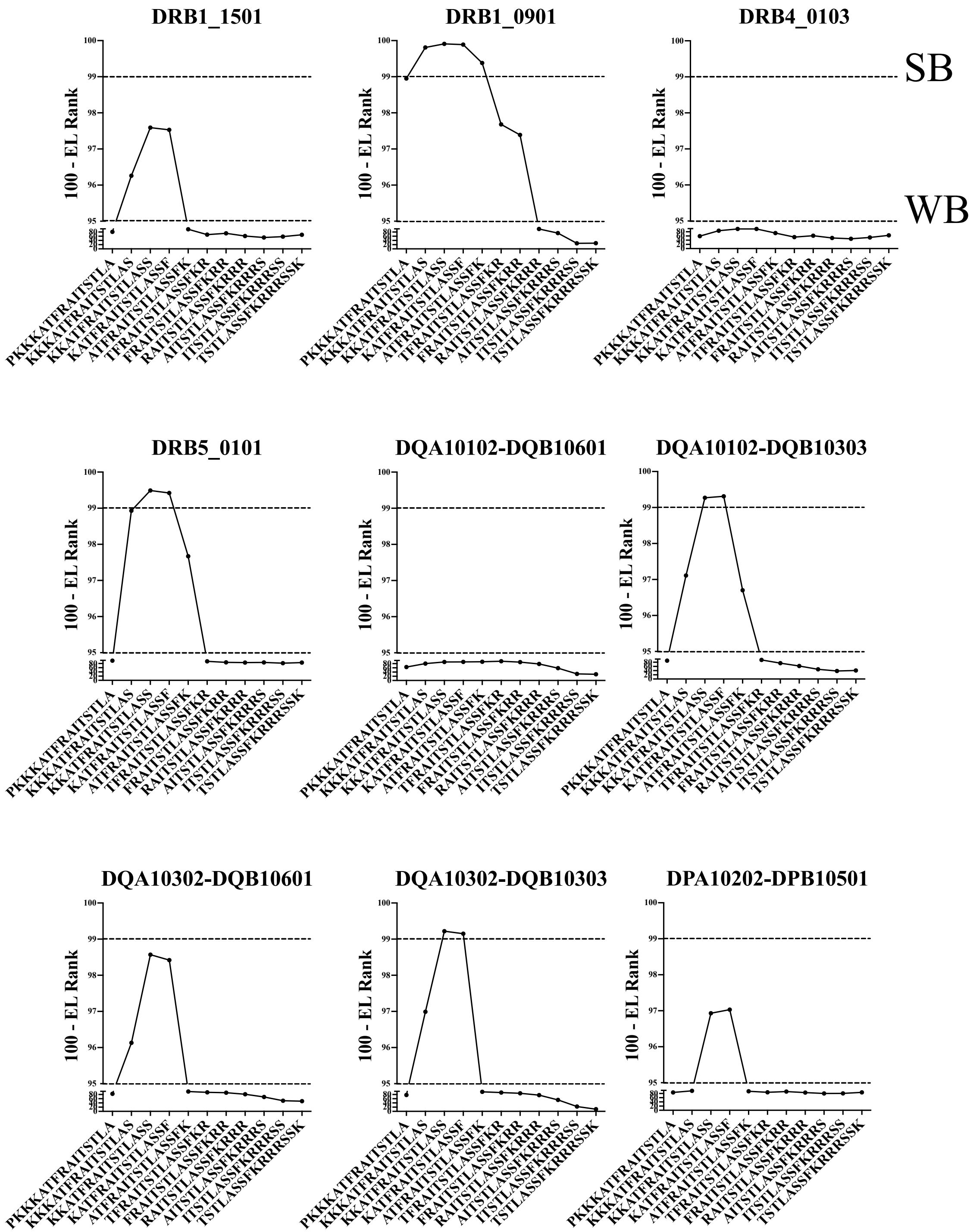

Supplementary figure 5: HLA binding predictions for identified immunogenic epitopes (class II). Class II prediction for CD4+ T cell epitope 2 in healthy volunteer 3. Binding predictions were made using NetMHCIIpan-4.0 using the identified epitopes and the known Human Leukocyte Antigen (HLA) molecules of the responding patients and healthy volunteers. Top dashed line represents cut off for strong binding (SB) criteria, bottom dashed line represents cut off for weak binding (WB) criteria. For visual presentation, peptide-MHC binding predictions for each peptide are presented using the bioinformatically calculated prediction score (% Rank EL) subtracted from 100% (used as the optimal score) i.e. 100 -%Rank_EL. EL = eluted ligand.
