# Supplementary material for: Incomplete T-cell tolerance and epitope-specific responses to the NR1 subunit in NMDA receptor antibody-associated encephalitis
Source: Brain Commun. 2026 Aug 5;8(4):fcag298. doi: 10.1093/braincomms/fcag298 (PMC13479385; doi:10.1093/braincomms/fcag298)
Supplement: Supplementary file 1 [file fcag298_supplementary_data.zip › Supplementary_Tables.pdf]

Supplementary table 1: Summary of demographics, clinical details and peptide pools eliciting responses at each round of peptide pool stimulation for patients and healthy volunteers tested

**Patient/ Healthy Volunteer**	**P1**	**P2**	**P3**	**P4**	**P5**	**P6**	**P7**	**P8**
**Age at sampling (decade)**	5th	4th	3rd	2nd	4th	2nd	4th	5th
**Clinical phenotype**	Seizures, sleep disturbance, autonomic disturbance	Neuropsychiatric symptoms, seizures, movement disorder	Neuropsychiatric symptoms	Neuropsychiatric symptoms, seizures, dysautonomia	Neuropsychiatric symptoms, movement disorder, dysautonomia	Neuropsychiatric symptoms, seizures, movement disorder, dysautonomia	Neuropsychiatric symptoms, seizures, movement disorder	Herpes simplex virus encephalitis, seizures, confusion
**Trigger**		Teratoma			Teratoma			HSVE
**CSF Ab at presentation**	Positive	Positive	Positive	Positive	Positive	Positive	Positive	Unknown
**Previous treatment given at point of sample collection**	None	Steroids, PLEX, rituximab, cyclophosphamide, bortezomib, teratoma resection	Steroids	Steroids, IVIG	Steroids, PLEX, teratoma resection	Steroids, PLEX, rituximab	Steroids, PLEX	None
**Months at sampling**	11	33	8	24	30	15	39	36
**Serum Ab at sample**	Positive	Negative	Negative	Positive	Negative	Positive	Positive	Positive
**Status at sampling**	Symptoms ongoing	Recovered	Recovered	Recovered, new symptoms	Recovered	Improving	Recovered	Recovered

Peptide pools eliciting responses	CD4+ d7	4,5,8,9,10	0	1,2,3,4,9	0	ND	0	1,2,4,9,10	0
	CD4+ d15	0	2,3,6	1,2,3,7	0	2,3,4,5	4,5	0	2
	CD4+ d23	0	0	0	0	6	0	10	2, 10
	CD8+ d7	10	0	1,2,4,5,6,8,9,10	0	ND	0	1,4,9	10
	CD8+ d15	4,8,9	1,3,4,6,8,9	6,8	0	2,3	0	1,5,8	2,5,8
	CD8+ d23	1,2,3,8,9	4,5,6,8	6,8,9,10	8	2,3,4,6,8	1,2,5,6,8	4,5,6	6,9,10
**Patient/ Healthy Volunteer**		**HV1**	**HV2**	**HV3**	**HV4**	**HV5**	**HV6**	**HV7**	**HV8**
**Age at sampling. (decade)**		5th	4th	4th	4th	3rd	4th	3rd	3rd
Peptide pools eliciting responses	CD4+ d7	ND	0	10	1	0	0	0	0
	CD4+ d15	1,9	4	10	3	3,4,5,6,8,9	4	0	0
	CD4+ d23	1,9	4,10	10	0	0	0	0	2,4,5,6,8,9
	CD8+ d7	ND	0	8	0	0	0	9	0
	CD8+ d15	1,10	6	3,8	1,3,4,5	6,9	1,2,3,4	1,9	1,2
	CD8+ d23	3,8,10	6,8,10	3,4,8,10	1,2,3,4,6,8,9	4,5,6	0	9	2,3,6,8

Supplementary table 2: Summary of identified CD4+ and CD8+ T cell epitopes to which responses were identified in either patient or healthy volunteer samples using matrix experiments.

CD8+ T cell epitope	NR1 amino acids	Peptide sequence	Patient or healthy volunteer
1	147-177	*QSSVWFEMMRVYSWNHIILLVSDDHEGRAAQ*	P1, P2, P5
2	403-33	*IHQEPFVYVKPTLSDGTCKEEFTVNGDPVKK*	P7
3	455-473	*CYGFCIDLLIKLARTMNFT*	P3, P8, HV8
4	499-513	*NGMMGELLSGQADMI*	HV5
5	557-583	*PFQSTLWLLVGLSVHVVAVMLYLLDRF*	P3
6	641-655	*MIIVASYTANLAAFL*	P3
7	745-760	*LVTTGELFFRSGFGI*	P1, P3, P5
8	774-792	*LSILKSHENGFMEDLDKTW*	P4, HV8
CD4+ T cell epitope	NR1 amino acids	Peptide sequence	Patient or healthy volunteer
1	455-469	*CYGFCIDLLIKLART*	P8, HV8
2	874-904	*PKKKATFRAITSTLASSFKRRRSSKDTSTGG*	P8, HV3
